# *Citrobacter braakii* bacteremia-induced septic shock after colonoscopy preparation with polyethylene glycol in a critically ill patient: a case report

**DOI:** 10.1186/s12941-017-0201-5

**Published:** 2017-04-04

**Authors:** Tetsuya Yumoto, Yoshiyasu Kono, Seiji Kawano, Chihiro Kamoi, Atsuyoshi Iida, Motoko Nose, Keiji Sato, Toyomu Ugawa, Hiroyuki Okada, Yoshihito Ujike, Atsunori Nakao

**Affiliations:** 1 Advanced Emergency and Critical Care Medical Center, Okayama University Hospital, 2-5-1 Kita-ku, Shikata-cho, Okayama-shi, Okayama, 700-8558 Japan; 2 Department of Gastroenterology and Hepatology, Okayama University Graduate School of Medicine, Dentistry and Pharmaceutical Sciences, 2-5-1 Shikata-cho, Kita-ku, Okayama-shi, Okayama, 700-8558 Japan; 3 Department of Clinical Laboratory, Okayama University Hospital, 2-5-1 Shikata-cho, Kita-ku, Okayama-shi, Okayama, 700-8558 Japan; 4 Department of Acute Care and Primary Care Medicine, Kawasaki Medical School Hospital, 577 Matsushima, Kurashiki-shi, Okayama, 701-0192 Japan

**Keywords:** Septic shock, *Citrobacter braakii*, Polyethylene glycol, Colonoscopy, Case report

## Abstract

**Background:**

Polyethylene glycol (PEG) is widely used for bowel cleaning in preparation for colonoscopy because of its safety. Septic shock after PEG preparation is an extremely rare complication. Herein, we describe a case of septic shock that occurred immediately after colonoscopy preparation with PEG.

**Case presentation:**

A 75-year-old Japanese male who had previously developed diabetes after total pancreatectomy received PEG in preparation for colonoscopy. He had been admitted to the emergency intensive care unit 4 days earlier due to hematochezia presenting with shock. He ingested PEG to prepare for a colonoscopy examination, which was performed to identify the source of his bleeding over a 5-h period, but suddenly exhibited septic shock and markedly elevated procalcitonin levels. A blood culture subsequently revealed *Citrobacter braakii*. Immediate resuscitation and intensive care with appropriate antibiotics improved his condition.

**Conclusions:**

Clinicians should be aware of the possibility of deteriorating conditions after bowel preparation with PEG among severely ill patients with recent episodes of hemorrhagic shock.

## Background

Polyethylene glycol (PEG) is widely used as a bowel preparation for colonoscopy because of its safety [1]. Only two cases of bacteremia after PEG ingestion have been reported, which were supposed to have been caused by bacterial translocation [2, 3]. Although *Citrobacter* spp. often causes opportunistic infections, *Citrobacter braakii* bacteremia infections are relatively uncommon [4, 5]. Herein, we describe a case of septic shock due to *C. braakii* bacteremia that occurred immediately after colonoscopy preparation with PEG in an adult patient who had recently suffered an episode of hemorrhagic shock.

## Case presentation

A 75-year-old Japanese male was transferred from another hospital after presenting with hemorrhagic shock due to hematochezia. He had been transported by ambulance to the previous hospital after complaining of discomfort and bloody stool on the previous day. He had undergone distal pancreatectomy and right lower lobectomy for pancreatic cancer and lung cancer, respectively, 5 years prior and total pancreatectomy due to residual pancreatic cancer eight months prior. The reconstruction procedure consisted of hepaticojejunostomy, Braun’s anastomosis, and gastrojejunostomy. In addition, a stent had been inserted for stenosis of the hepaticojejunostomy 1 month prior. The patient’s medical history also included atrial fibrillation, and he was taking insulin and apixaban. On arrival, he appeared to be pale and his extremities were cold. His vital signs were as follows: respiratory rate, 20 breaths/min; pulse rate, 105 beats/min (bpm); blood pressure, 77/54 mmHg; temperature, 34.9 °C. Arterial blood gas analysis detected severe lactic acidosis (lactate concentration: 9.9 mmol/L) and anemia (hemoglobin level: 3.1 g/dL). The laboratory data showed a white blood cell count of 5850/μL, C-reactive protein level of 0.08 mg/dL, procalcitonin level of 0.097 ng/mL, and serum glucose level of 389 mg/dL. Contrast-enhanced computed tomography of the abdomen did not detect any active bleeding. His melena had already ceased. Fluid resuscitation and massive transfusions resolved his hemorrhagic shock. Crystalloids were administered 800 mL over an hour. Blood products were initiated 30 min after the patient’s arrival. The patient required 16 units of red blood cell concentrate, 14 units of fresh frozen plasma and 20 units of platelets within 12 h of his arrival for hemostatic resuscitation. A classification of hemorrhage of the patient was considered as class IV based on the American College of Surgeons. Urgent upper gastrointestinal endoscopy was a poor study because of food residue and failed to identify the source of the patient’s bleeding. Thus, he was admitted to the emergency intensive care unit for careful observation. The patient’s acute physiology and chronic health evaluation (APACHE II) score on the day of admission was 24, and the Charlson comorbidity index was three.

Since fresh bleeding was detected in the terminal ileum during capsule endoscopy the next day, colonoscopy was performed without bowel cleaning. But the exam produced poor findings due to blood clot. Therefore, transanal double balloon enteroscopy was scheduled for further investigation 4 days after the patient’s admission. The patient took 5 h to ingest 2 L of PEG plus an additional liter of PEG for bowel preparation. This was the first time he had consumed PEG. 1 h after consuming the 3 L of PEG, he complained of a feverish chill and his heart rate and temperature increased to over 130 bpm and 39 °C, respectively. The transanal double balloon enteroscopy exam was performed uneventfully and did not reveal the source of the patient’s bleeding.

**Fig. 1:**
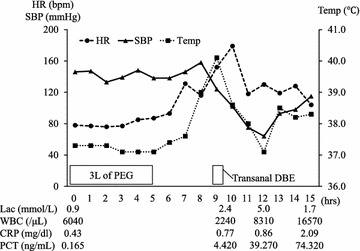
Patient’s clinical course during the 15 h after he consumed PEG. *PEG* polyethylene glycol, *HR* heart rate, *SBP* systolic blood pressure, *temp* temperature, *DBE* double balloon enteroscopy, *lac* lactate, *WBC* white blood cell count, *CRP* C-reactive protein, *PCT* procalcitonin

**Fig. 2:**
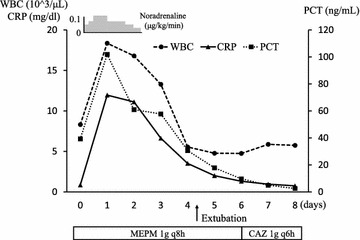
The patient’s clinical course over the 8 days after the onset of septic shock. *WBC* white blood cell count, *CRP* C-reactive protein, *PCT* procalcitonin, *MEPM* meropenem, *CAZ* ceftazidime

After the examination, he appeared to be agitated and distressed and exhibited hypotension, high fever, and an elevated lactate level, which indicated septic shock. He did not have any other complaints. His abdomen was soft, flat, and non-tender. Empirical antibiotic therapy with meropenem was administered, and two sets of blood cultures were obtained at the same time. Fluid resuscitation and a noradrenaline infusion were initiated due to septic shock, followed by intubation and mechanical ventilation. Table 1 shows the patient’s laboratory data at the time that he suffered septic shock. Repeated lab tests revealed a significantly elevated procalcitonin level. Liver function tests produced normal results during the course, so we excluded biliary stent infection. Figure 1 shows the patient’s clinical course during the first 15 h after he ingested PEG, the hypotensive phase of his condition, and the resuscitation period. Ongoing intensive care led to a gradual improvement in the patient’s condition and he was successfully extubated on day 4 (the day when the PEG preparation was administered was defined as day 0, at which APACHE II score was 34). An examination of his blood cultures detected *C. braakii* and we replaced the meropenem with ceftazidime based on the results of sensitivity tests. Later, the identity of the pathogen was confirmed by biochemical analysis and partial sequencing of 16S rRNA. Urinalysis produced normal results. Cultures of the patient’s urine and sputum at the onset of septic shock were negative. The tip cultures of central venous catheter which had been placed in a femoral vein on admission were found to be negative. Stool culture was obtained 1 week after presenting septic shock, which was also negative. Antibiotics were administered for a total of 10 days. The patient was discharged from the emergency intensive care unit on day 8. Figure 2 shows the patient’s clinical course over the 8 days after he first suffered septic shock.

**Table 1:** The patient’s laboratory data at the time when he exhibited septic shock

Arterial blood gas analysis (3L/min of oxygen)		Complete blood cell count		Biochemistry		Coagulation	
pH	7.431	WBC	8310/μL	TP	5.6 g/dL	PT	56%
PaCO_2_	30.6 mmHg	NE	97.9%	Albumin	3.1 g/dL	INR	1.31
PaO_2_	93.5 mmHg	LY	1.1%	AST	20 U/L	APTT	28 s
HCO_3_^−^	20 mmol/L	MONO	0.4%	ALT	9 U/L	Fibrinogen	181 mg/dL
Base excess	−3.2 mmol/L	Hgb	9.2 g/dL	T-Bil	2.08 mg/dL	D-dimer	6.1 μg/mL
Lactate	5 mmol/L	Hct	27.1%	D-Bil	0.83 mg/dL	AT3	58%
Glucose	200 mg/dL	PLT	9.6 × 10^4^/μL	BUN	17.3 mg/dL		
				Creatinine	1.20 mg/dL		
				Na	136 mmol/L		
				K	3.4 mmol/L		
				Cl	106 mmol/L		
				CRP	0.86 mg/dL		
				Procalcitonin	39.27 ng/mL		
				Endotoxin	≤0.8 pg/mL		

*WBC* white blood cell count, *NE* neutrophils, *LY* lymphocytes, *MONO* monocytes, *Hgb* hemoglobin, *Hct* hematocrit, *PLT* platelet count, *PT* prothrombin time, *INR* international normalized ratio, *APTT* activated partial thromboplastin time, *AT3* antithrombin 3, *TP* total protein, *AST* aspartate transaminase, *ALT* alanine transaminase, *T*-*Bil* total bilirubin, *D*-*Bil* direct bilirubin, *BUN* blood urea nitrogen, *Na* sodium, *K* potassium, *Cl* chlorine, *CRP* C-reactive protein

Melena occurred intermittently. Upper gastrointestinal endoscopy subsequently identified marginal ulceration of the gastrojejunal anastomosis, which was successfully treated with coagulation hemostasis. The patient was discharged on day 33.

## Discussion

The course of this patient provided two important clinical implications. First, PEG for bowel preparation can possibly induce septic shock in critically ill patients. Second, *C. braakii*, which is an unusual pathogen of sepsis, can be responsible for septic shock.

There have only been two previously reported cases of bacteremia after PEG ingestion. Fukutomi et al. reported a 58-year-old male with a history of ulcerative colitis who developed septic symptoms 2 h after consuming PEG [2]. He subsequently suffered an intervertebral canal abscess. *Escherichia coli* was isolated from the patient’s feces and peripheral blood. It was speculated that bacterial translocation might have occurred after the patient ingested PEG. Darrow et al. reported an 8-year-old boy who suffered *E. coli* bacteremia during the treatment of functional constipation via PEG-based fecal disimpaction [3]. They concluded that physical damage to the intestinal mucosa had contributed to the occurrence of bacterial translocation.

The possible infection route of the present case is discussed. The time sequence during which the patient abruptly deteriorated soon after ingesting PEG was suggestive of septic shock induced by PEG. Unremarkable urinalysis and liver function test findings, as well as negative urine and sputum culture’ results, excluded urinary, respiratory, and biliary tract infections. The negative results of central venous catheter tip cultures excluded catheter-related bloodstream infection. As for transfusion, Hauser et al. reported a fatal case of transfusion-transmitted infection due to *C. koseri*. The patient developed sepsis only 30 min after the start of the transfusion [6]. While, our patient required blood products only on the day of admission, which was considered less likely to have been infected via transfusion. Although there was no evidence supporting bacterial translocation, it could not be completely denied because of the difficulties in detecting bacterial translocation in humans [7]. In addition, prior antibiotic therapy might have led to negative stool culture results, which were obtained one week after the onset of septic shock. Nevertheless, the stool culture result was not necessarily consistent with pathogenic microbes [8]. In the present case, gastrointestinal tract might have been a possible portal of entry, although there was no clear evidence.

*Citrobacter*. *braakii*, an unusual pathogen of sepsis, can be responsible for septic shock. *Citrobacter* spp. are Gram-negative bacilli that can cause septicemia mainly in immunocompromised patients with conditions such as cancer, alcoholism, diabetes, and congestive heart failure [4]. In a previous study on the *Citrobacter* infections encountered at a tertiary university hospital, Samonis et al. reported that the most common causative organism of septicemia was *C. freundii* (71.8%), followed by *C. koseri* (23.1%) and *C. braakii* (3.8%), and the most common types of infection were urinary tract infections (52.6%), followed by intra-abdominal (14.1%), surgical site (7.7%), skin and soft tissue (6.4%), and respiratory tract infections (6.4%) (5). Septicemia due to *C. braakii* is extremely rare, and only three cases have been reported, which involved lower extremity cellulitis in a renal transplant patient receiving immunosuppressant therapy, and acute peritonitis in a peritoneal dialysis patient and in a patient with cervical cancer [6, 9, 10]. In the present case, the recent episode of hemorrhagic shock may have resulted in an immunocompromised condition. In addition, diabetes and a history of lung and pancreatic cancer may have also contributed to the condition.

To the best of our knowledge, this is the first report about *C. braakii* bacteremia-induced septic shock after PEG ingestion for colonoscopy preparation. Several limitations should be addressed including inability to generalize the results and inadequate evidence of a cause-effect relationship due to its nature of the case report. In contrast, generating hypotheses and educational value should be acknowledged as the strengths of the case report. Further reports should be published to determine whether septic shock induced by PEG ingestion in such an unstable condition may be much more frequently present. Nevertheless, physicians should be aware of patient deterioration after bowel preparation under these predisposing conditions.

## Conclusions

We reported a case of *C. braakii* bacteremia-induced septic shock that occurred immediately after PEG ingestion for colonoscopy preparation. Clinicians should be aware of the life-threatening complications of bowel preparation with PEG in such critically ill patients.

## Abbreviations

PEG: polyethylene glycol
APACHE II: acute physiology and chronic health evaluation
